# Measurement of Service Quality Gaps in Dental Services using SERVQUAL in Public Hospitals of Rawalpindi

**DOI:** 10.12669/pjms.37.3.3436

**Published:** 2021

**Authors:** Asmaa Riaz, Ume Sughra

**Affiliations:** 1 Dr. Asmaa Riaz, BDS, MSPH., Al-Shifa School of Public Health, Al-Shifa Trust Eye Hospital, Rawalpindi, Pakistan; 2 Dr. Ume Sughra, MBBS, MPH, FCPS. Associate Professor of Community Medicine & Research Associate, Al-Shifa School of Public Health, Al-Shifa Trust Eye Hospital, Rawalpindi, Pakistan

**Keywords:** Dental services, Service Quality, SERVQUAL, Expectations, Perceptions, Public Hospitals

## Abstract

**Objectives:**

To measure service quality gaps in dental services provided at public hospitals of the district, Rawalpindi.

**Methods:**

A cross-sectional survey was conducted in two of the public hospitals of the district, Rawalpindi from April to October 2019. Non-probability consecutive sampling was used to include a total number of 400 patients, equally divided between Rural health center (RHC) and Tehsil headquarter (THQ). Face to face interviews were done using a 32-item SERVQUAL in the form of a structured questionnaire where one part of the questionnaire was filled before the treatment and the other after the treatment. Cronbach’s alpha coefficient was found to be 0.90. It was analyzed using SPSS version 25 with descriptive and parametric tests, and further multiple linear regression was done.

**Results:**

The quality of services provided to patients was significantly lower than their expectations in both RHC (-14.48 ± 7.96) and THQ (-9.97 ± 7.97). Independent t-test showed a significant difference in service quality between both the hospitals (-4.41), with a better quality of services in THQ. Association of service quality gap was statistically significant with gender, education, occupation, monthly income, and the number of visits to the hospital with p-value < 0.05. The type of hospital was the strongest predictor (ß = 4.12) of the outcome variable.

**Conclusion:**

The findings reveal that patients’ expectations exceed their perception of dental services provided in public hospitals. THQ provided a better quality of services compared to RHC.

## INTRODUCTION

With the advancement and growing competition, provision of quality services has become the main objective of service providers including healthcare.^1^ Quality in the healthcare system consists of technical quality and functional quality. Technical quality refers to technical accuracy of healthcare providers ’ diagnosis and procedure, while functional quality refers to the way health services are provided to the patients.^2^ Health care providers generally focus on functional quality to assess the services, which patients can evaluate as they are based on their experience of the services, whereas technical aspects require medical expertise and knowledge.^3^

SERVQUAL is a widely used tool developed to measure quality of services. It consists of five dimensions (tangibility, reliability, responsiveness, empathy and assurance) that was developed to be used in the marketing industry, but with slight modification, it can be used by any organization because of its comprehensiveness and practical applicability.^4^

Service quality is measured through expectations and perceptions of customers where “Perceptions refer to the consumers’ evaluation of the services provided and expectations are viewed as desires or wants of consumers, i.e., what they feel a service provider should offer rather would offer.” The difference between them along the quality dimensions determines the service quality gap.^4^ The tool has also been widely used in healthcare to evaluate its services.^5^

The increased costs of dental treatment and with patients being more aware, having access to information has increased the demands for quality services. According to the Organization for Economic Co-operation and Development (OECD), dental treatment is expensive even in high-income countries, accounting for 5% of total health expenditure and 20% of out-of-pocket health expenditure.^6^ The access to dental services provided by the public hospitals in Pakistan starts from rural health centers although basic health units are the first level of care facilities. There are 638 RHC in total that are expected to provide curative services to about 64 percent of the population, which means one dentist for a population of nearly 200,000 people.^7^ Both secondary and tertiary hospitals are also limited to curative services due to overload of patients from rural areas.^8^

The growing need and competition from the private sector has added additional pressure on the public sector to justify its existence as organizations offering essential services of the highest quality.^9^ This study focuses on measuring the quality of services from a patient’s point of view.

## METHODS

This cross-sectional study was conducted from April to October 2019 in one primary and one secondary health care center of Rawalpindi, Pakistan. The study population were the patients receiving dental services in primary and secondary hospitals of district Rawalpindi. A sample of 400 respondents was taken, calculated by using the formula: z^2^* p * q/e^2^ with a proposed proportion of 50% satisfaction, at 95% confidence level. It was equally divided between the RHC and THQ and patients were selected through non-probability consecutive sampling. The study was conducted after getting the approval from the Ethical Review Committee of Pakistan Institute of Ophthalmology, Al-Shifa School of Public Health (Reference No.: ERC-50/AST-I9, Dated: 6^th^ May, 2019). All patients aged above 18 years, both male and female who had dental treatment done in the hospitals (THQ and RHC) on the days of data collection were included in the study. Patients who had any procedure done in private facilities, had severe dental complications, or dental trauma or those not willing to participate were excluded. Informed consent was taken verbally from all patients before data collection.

Data were collected using a pre-tested structured questionnaire, first developed in English, then later translated into Urdu, the national language of Pakistan. A self-administered questionnaire was constructed but due to the low level of literacy for most of the patients face to face interviews were conducted; others preferred to fill out the questionnaire by themselves. It was validated by carrying out a pilot survey, and changes were made accordingly. Reliability analysis was done and Cronbach’s alpha coefficient was found to be 0.90.

The first section consisted of demographic characteristics of the patients and the second and third sections contained 16 matching statements for expectation (taken before treatment) and perception (taken after the treatment), evaluating service quality. Each statement was assessed on a 5 point Likert type scale (1- Strongly disagree to 5- Strongly agree). It considered five service quality dimensions: tangibles, reliability, responsiveness, assurance, and empathy.

Data entry and statistical analysis were done using SPSS software version 25. The outcome variable was the service quality gap, calculated by computing the patient’s responses of perception and expectations. Paired t-test was used to calculate the mean score difference between expectation and perception of SERVQUAL dimensions, and Independent Samples T-Test to compare service quality between RHC and THQ. Association of service quality with different demographic factors was computed using T-test and One-Way ANOVA. Further, multiple linear regression was done to check for predictors of service quality.

## RESULTS

Of the 400 patients, 236 (59%) were females and the predominant age group was 18-35 years around 194 (49%). Three hundred and forty-six (86%) were married and 152 (38%) were uneducated. Around two hundred and twenty-one (55%) of the patients were unemployed, and 76 (19%) were self- employed. Patients with monthly income ranged between 10,000 to 20,000 PKR were 93(24%) and about 295 (74%) reported that the hospital was at a convenient location. About 257 (64%) had previously visited the hospital, and the highest number of previous visits was once 106 (27%). There were 231 (58%) who chose the hospital for treatment charges and 94 (24%) for its services with 203 (51%) of the patients came due to dental pain (Table-I).

**Table-I:** Association of demographic characteristics with Service Quality.

Variables	No.	%	test	p – value
Age**				
18 - 35	194	49		
36 - 55	151	38	3.09	0.04
56 - 75	48	12		
75+	07	2		
Gender*				
Male	164	41	-2.99	0.003
Female	236	59		
Marital Status*				
Married	346	87	2.41	0.016
Unmarried	54	13		
Education**				
Uneducated	152	38	4.13	0.001
Primary Education	39	10		
Matric	140	35		
Intermediate	55	14		
Graduation	08	02		
Post-Graduation	06	01		
Occupation**				
Unemployed	221	55	4.62	0.001
Self employed	76	19		
Government Job	14	04		
Private Job	28	07		
Labor	46	11		
Retired	15	04		
Monthly Income (PKR)**				
Less than 10,000	33	08	2.57	0.03
10,000 – 20,000	93	24		
21,000 – 30,000	50	13		
More than 30,000	08	2		
Not applicable	216	54		
Convenient Location*				
No	105	26	-0.66	0.52
Yes	295	74		
Visits to hospital**				
First Visit	144	36	4.99	0.002
Second Visit	107	27		
Third Visit	93	23		
More than three visits	56	14		
Preference for the hospital**				
Referred	02	1	0.44	0.77
Charges	94	23		
Services	231	57		
Skilled Staff	1	1		
Skilled Doctor	72	18		
Reason for the visit**				
Scaling	14	3	2.10	0.08
Dental pain	203	51		
Tooth Extraction	134	33		
Filling	42	11		
Root Canal Treatment	07	2		

* Independent T test,

** One Way Anova

The highest expectations of patients in both RHC and THQ were that the dentist should deal with them in a caring fashion. The best perception in RHC was for the way the dentist dealt with them, and for THQ, the highest perceptions were that the dentist was well mannered (Table-II).

**Table-II:** The expectations and perceptions of patients in RHC and THQ for dental services.

DIMENSIONS	Expectations		Perceptions	

	RHC	THQ	RHC	THQ

	Mean ± SD	Mean ± SD	Mean ± SD	Mean ± SD
***Tangibles***				
Hospital have up to date equipment’s.	3.67 ± 0.47	3.32 ± 0.46	1.92 ± 1.00	2.89 ± 0.60
Hospital have comfortable waiting area.	3.52 ± 0.50	3.30 ± 0.45	2.94 ± 0.48	2.91 ± 0.88
Medicines are easily available in the hospital.	3.50 ± 0.50	3.39 ± 0.49	2.84 ± 0.52	3.04 ± 0.68
***Reliability***				
Hospital provides services as promised.	3.50 ± 0.50	3.31 ± 0.46	1.91 ± 1.00	2.84 ± 0.75
Hospital provides services on time	3.51 ± 0.50	3.30 ± 0.45	2.41 ± 0.89	2.41 ± 1.06
Hospital has convenient operating hours.	3.51 ± 0.50	3.30 ± 0.45	2.29 ± 0.99	2.25 ± 1.11
***Responsiveness***				
Staff provides the best services possible.	3.51 ± 0.50	3.30 ± 0.45	2.65 ± 0.71	2.38 ± 0.96
Staff is willing to help their patients.	3.51 ± 0.50	3.30 ± 0.45	2.72 ± 0.49	2.61 ± 0.83
Staff shows sincere interest to solve patient’s problems.	3.72 ± 0.45	3.61 ± 0.48	2.68 ± 0.50	2.73 ± 0.74
***Empathy***				
Staff gives individual attention to patients.	3.53 ± 0.50	3.30 ± 0.45	2.67 ± 0.51	2.82 ± 0.76
Staff understands patient’s specific needs.	3.51 ± 0.50	3.30 ± 0.45	3.02 ± 0.14	2.75 ± 0.72
Dentist should do his best to make treatment pain free.	3.75 ± 0.43	3.70 ± 0.45	3.04 ± 0.59	2.93 ± 0.61
***Assurance***				
Dentist is well mannered.	3.75 ± 0.43	3.69 ± 0.46	2.94 ± 0.42	3.24 ± 0.62
Dentist deals in a caring fashion.	3.76 ± 0.42	3.76 ± 0.46	3.09 ± 0.52	3.22 ± 0.62
Dentist should assure regarding the better treatment option.	3.76 ± 0.42	3.76 ± 0.45	3.07 ± 0.57	3.08 ± 0.91
Dentist should be knowledgeable to answer any question.	3.75 ± 0.43	3.75 ± 0.45	3.08 ± 0.57	3.12 ± 0.86

**Fig.1:**
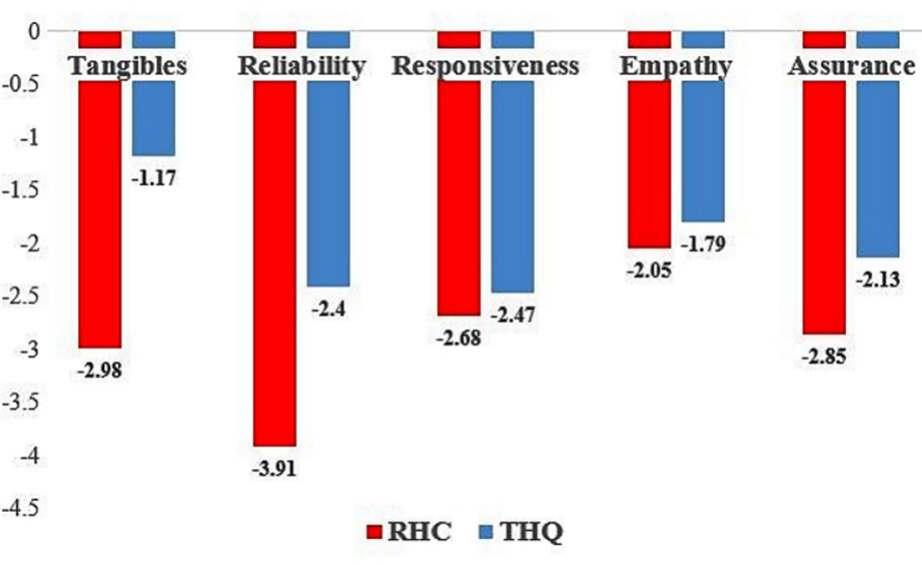
Comparison of service quality gap score in different dimensions of SERVQUAL between the Tehsil Headquarter and Rural Health Center.

Paired T test depicted the highest Service Quality gap score in RHC for reliability (-3.91 ± 2.25) followed by tangibility (-2.98 ± 1.79) whereas in THQ the highest gap score was for responsiveness (-2.47 ± 2.26) followed by reliability (-2.40 ± 2.03). There was statistically significant difference between perceptions and expectations in all the dimensions in both RHC and THQ [t (199) = -25.7; p = 0.005], [t (199) = -23.88; p = 0.005] respectively. On comparing Service Quality gap scores using Independent T test, a statistically significant difference was found between RHC (-14.48 ± 7.96) and THQ (-9.97 ± 7.97); [t (398) = -5.66; p = 0.005]. The magnitude of the difference in the means (4.41; 95% CI = -6.02 to-2.94) was moderate with effect size 0.07 (Fig.1).

Associations with age, gender, marital status, education, occupation, monthly income, and visit to the hospital were also found to be significant with a p-value less than 0.05 (Table-II). From 11 independent variables, type of hospital, age, and education were found to have significant variance where the type of hospital was the strongest predictor (ß = 4.12). Overall model was statistically significant [F= 6.74; P value=0.0005], and the variables were responsible for 16% variation in outcome variable.

## DISCUSSION

For any health care organization, it has become a necessity to continuously evaluate their services for progress and survival in today’s competitive world.^10^ A comparative analysis of dental services between primary (RHC) and secondary healthcare (THQ) was done to identify the key factors and areas, providing guidance to improve the quality of services. The main findings of the study showed that the quality of services provided to patients was significantly lower than their expectations in both RHC (-14.48 ± 7.96) and THQ (-9.97 ± 7.97). There was also a significant difference in service quality between both the hospitals (-4.41) with p-value < 0.05.

Patients in both RHC (3.76 ± 0.42) and THQ (3.76 ± 0.46) had the highest expectations for the way the dentist should deal with them. Dentist-patient interaction is considered one of the most significant aspect of a dental visit.^11^ The lowest expectations in RHC were for the dental services that had been promised (3.50 ± 0.50). In THQ, the lowest expectations (3.30 ± 0.45) were for a hospital having convenient operating hours for dental services. According to a study done in public hospitals, patients were satisfied with the expertise of the doctor.^12^ These results are in conjunction with our study, where both the hospitals showed the highest perceptions for assurance (RHC: 12.17 ± 1.84; THQ: 12.65 ± 2.93) compared to other dimensions, despite high expectations of patients. This indicates that dentists are doing their best to satisfy patients with the limited services that they can offer despite the non-availability of materials or nonfunctional equipment.

Significant differences between all dimensions of service quality were reported in these studies.^13^-^16^ Our study reported results in accordance with this study, where both the hospitals showed significant differences in expectations and perceptions for all the dimensions of service quality. In this study, the highest gap was in for RHC was in reliability (-3.91 ± 2.25) whereas in THQ highest gap was for responsiveness (-2.47 ± 2.26). This result was similar to a study where reliability was reported with the highest quality gap.^14^,^17^ The high score in this study for the dimension reliability is due to the non-availability of dental materials in RHC, which results in less number of services available, or it delays the treatment process. The patients and the dentists both prefer for extraction leading to more number of extractions carried out in public hospitals compared to restorative treatments. This is supported by the evidence in this study, where 33% came for extraction and only 2% for root canal treatment. The high gap score in reliability for both hospitals is explained by the fact that all public hospitals lack dental rehabilitative services. Further, in public hospitals, dental services are available only during morning hours that are from 8-AM to 2-PM, coinciding with office/working hours making it difficult for patients to receive any care. Those having any dental issues during evening times are only medicated by medical officers on duty in general OPD. On comparing the service quality gap of THQ and RHC, a significant difference (-4.51) was found between both the hospitals. The highest gap was reported for tangibility (-1.81, p< 0.05) and reliability (-1.51, p< 0.05). More number of patients in THQ 50% were found to be satisfied with services compared to RHC.

In this study, a significant association of service quality gap was found with gender, education, occupation, monthly income, and the number of visits to the hospital (p < 0.05). Statistically significant influence of different demographic characteristics on service quality was also reported in these studies.^15^,^18^ A significant association was reported between gender and the quality gap so that the gap was higher in females than males.^19^ This finding is contrary to our study, where the gap was higher in males (-13.70 ± 8.22) than females (-11.20 ± 8.17). The significant predictors for outcome variable based on the findings of this study were the hospital, age, gender, and education. Among these predictors, the type of hospital was the strongest predictor (ß = 4.28). Multiple regression analysis in another study showed that all independent variables influenced satisfaction, except age and marital status.^20^

### Limitation of the study:

Due to time constraints, it was conducted in one of the RHC and THQ of the district Rawalpindi. To enhance the generalizability of the findings, future studies should consider more number of public hospitals as well as private hospitals.

## CONCLUSION

The findings reveal that patients’ expectations exceeded their perception in all dimensions for dental services provided in both public hospitals. THQ provided a better quality of services compared to RHC.
